# 1-Iodo-4-meth­oxy-2-nitro­benzene

**DOI:** 10.1107/S1600536812006484

**Published:** 2012-02-17

**Authors:** James L. Wardell, Edward R. T. Tiekink

**Affiliations:** a Centro de Desenvolvimento Tecnológico em Saúde (CDTS), Fundação Oswaldo Cruz (FIOCRUZ), Casa Amarela, Campus de Manguinhos, Avenida Brasil 4365, 21040-900 Rio de Janeiro, RJ, Brazil; b Department of Chemistry, University of Malaya, 50603 Kuala Lumpur, Malaysia

## Abstract

In the title compound, C_7_H_6_INO_3_, the 12 non-H atoms are planar, with an r.m.s. deviation of 0.016 Å. A close intra­molecular I⋯O inter­action [3.0295 (13) Å] is present. Inter­molecular I⋯O inter­actions [3.3448 (13) Å] lead to the formation of zigzag chains along the *b* axis. These are assembled into layers by weak π–π inter­actions [centroid–centroid distance = 3.8416 (9) Å], and the layers stack along the *a* axis, being connected by C—H⋯O contacts.

## Related literature

For general background to halogen bonding, see: Metrangolo *et al.* (2008 ▶); Pennington *et al.* (2008 ▶). For previous structural studies probing iodo–nitro inter­actions, see: Glidewell *et al.* (2002 ▶, 2004 ▶); Garden *et al.* (2002 ▶). For van der Waals radii, see: Bondi (1964 ▶).
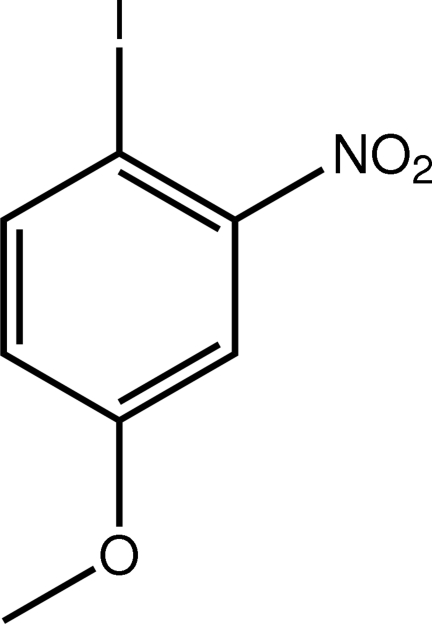


## Experimental

### 

#### Crystal data

C_7_H_6_INO_3_
*M* *_r_* = 279.03
Orthorhombic, 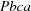

*a* = 18.6370 (13) Å
*b* = 11.6257 (5) Å
*c* = 7.4740 (3) Å
*V* = 1619.38 (15) Å^3^
*Z* = 8
Mo *K*α radiation
μ = 3.92 mm^−1^
*T* = 100 K
0.13 × 0.09 × 0.01 mm

#### Data collection

Rigaku Saturn724+ (2 × 2 bin mode) diffractometer
Absorption correction: multi-scan (*CrystalClear-SM Expert*; Rigaku, 2011 ▶) *T*_min_ = 0.755, *T*_max_ = 1.000
8053 measured reflections
1841 independent reflections
1615 reflections with *I* > 2σ(*I*)
*R*_int_ = 0.017

#### Refinement

*R*[*F* ^2^ > 2σ(*F* ^2^)] = 0.014
*wR*(*F* ^2^) = 0.039
*S* = 1.04
1841 reflections
110 parameters
H-atom parameters constrained
Δρ_max_ = 0.42 e Å^−3^
Δρ_min_ = −0.25 e Å^−3^

### 

Data collection: *CrystalClear-SM Expert* (Rigaku, 2011 ▶); cell refinement: *CrystalClear-SM Expert*; data reduction: *CrystalClear-SM Expert*; program(s) used to solve structure: *SHELXS97* (Sheldrick, 2008 ▶); program(s) used to refine structure: *SHELXL97* (Sheldrick, 2008 ▶); molecular graphics: *ORTEP-3* (Farrugia, 1997 ▶) and *DIAMOND* (Brandenburg, 2006 ▶); software used to prepare material for publication: *publCIF* (Westrip, 2010 ▶).

## Supplementary Material

Crystal structure: contains datablock(s) global, I. DOI: 10.1107/S1600536812006484/hg5176sup1.cif

Structure factors: contains datablock(s) I. DOI: 10.1107/S1600536812006484/hg5176Isup2.hkl

Supplementary material file. DOI: 10.1107/S1600536812006484/hg5176Isup3.cml

Additional supplementary materials:  crystallographic information; 3D view; checkCIF report

## Figures and Tables

**Table 1:** Hydrogen-bond geometry (Å, °)

*D*—H⋯*A*	*D*—H	H⋯*A*	*D*⋯*A*	*D*—H⋯*A*
C7—H7*B*⋯O1^i^	0.98	2.58	3.4503 (19)	148

Symmetry code: (i) 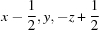.

**Fig. 1.**
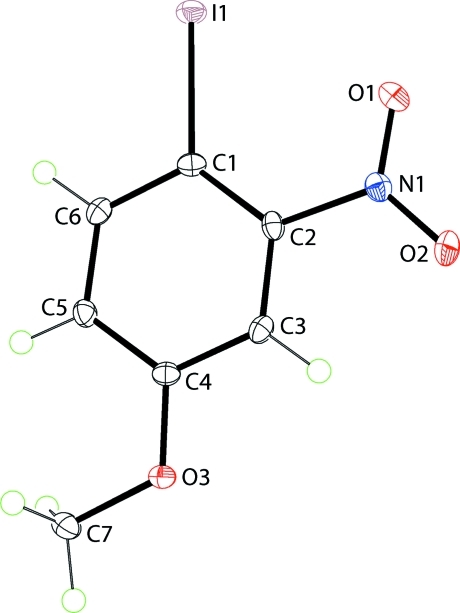
The molecular structure of (I) showing the atom-labelling scheme and displacement ellipsoids at the 50% probability level.

**Fig. 2.**
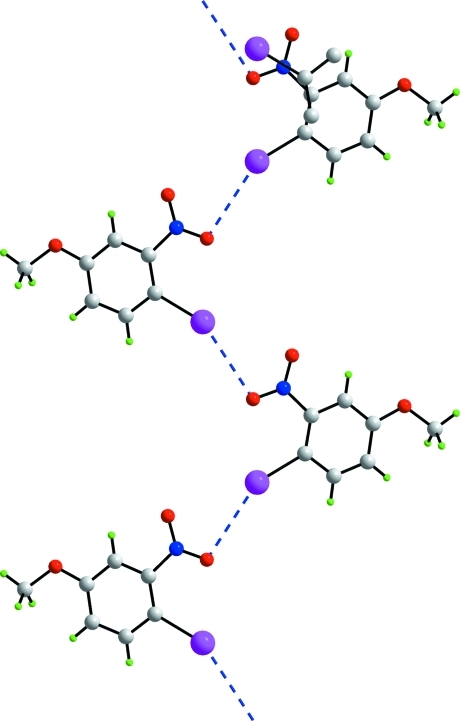
A view of the linear supramolecular chain along the b axis in (I). The I···O interactions are shown as blue dashed lines.

**Fig. 3.**
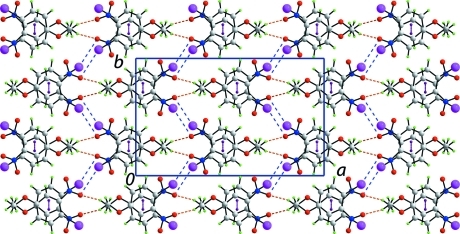
A view in projection down the c axis of the packing of supramolecular chains in (I). The I···O, C—H···O and π–π interactions are shown as blue, orange and purple dashed lines, respectively.

## supplementary crystallographic information

### Comment

In connection with previous structural studies of iodo···nitro interactions (Glidewell *et al.*, 2002; Garden *et al.*, 2002; Glidewell *et al.*, 2004), the crystal and molecular structure of the title compound (I) was undertaken in order to probe the structure for possible I···O halogen bonding (Metrangolo *et al.*, 2008; Pennington *et al.*, 2008).

The 12 non-hydrogen atoms comprising (I), Fig. 1, are co-planar with a rm.s. deviation = 0.016 Å; the maximum deviations of ±0.026 Å being found for the nitro-O atoms. A close intramolecular I1···O1 interaction of 3.0295 (13) Å is noted that is significantly less than the sum of the van der Waals radii for these atoms, *i.e*. 3.50 Å (Bondi, 1964). There are also notable intermolecular I1···O interactions, the shortest of 3.3448 (13) Å occurs with the O1^i^ atom [symmetry operation *i*: 3/2 - *x*, 1/2 + *y*, *z*]. A longer interaction [3.4530 (13) Å] is formed with the O2 atom of the same nitro group. The I1···O1^i^ interactions lead to the formation of zigzag chains along the *b* axis, Fig. 2. The supramolecular chains are assembled into layers in the *bc* plane by weak π–π interactions [ring centroid···centroid distance = 3.8416 (9) Å for symmetry operation *x*, 3/2 - *y*, -1/2 + *z*]. Layers stack along the *a* axis and are connected by C—H···O contacts, Fig. 3 and Table 1.

### Experimental

The commercial compound (Aldrich) was recrystallized from EtOH; *M*.pt: 334–335 K.

### Refinement

The *C*-bound H atoms were geometrically placed (C—H = 0.95–0.98 Å) and refined as riding with *U*_iso_(H) = 1.2–1.5*U*_eq_(C).

### Crystal data

**Table:**

C_7_H_6_INO_3_	*F*(000) = 1056
*M**_r_* = 279.03	*D*_x_ = 2.289 Mg m^−^^3^
Orthorhombic, *P**b**c**a*	Mo *K*α radiation, λ = 0.71073 Å
Hall symbol: -P 2ac 2ab	Cell parameters from 6840 reflections
*a* = 18.6370 (13) Å	θ = 3.4–27.4°
*b* = 11.6257 (5) Å	µ = 3.92 mm^−^^1^
*c* = 7.4740 (3) Å	*T* = 100 K
*V* = 1619.38 (15) Å^3^	Plate, dark-orange
*Z* = 8	0.13 × 0.09 × 0.01 mm

### Data collection

**Table:**

Rigaku Saturn724+ (2x2 bin mode) diffractometer	1841 independent reflections
Radiation source: Rotating Anode	1615 reflections with *I* > 2σ(*I*)
Confocal monochromator	*R*_int_ = 0.017
Detector resolution: 28.5714 pixels mm^-1^	θ_max_ = 27.5°, θ_min_ = 3.4°
profile data from ω–scans	*h* = −15→24
Absorption correction: multi-scan (*CrystalClear*-SM Expert; Rigaku, 2011)	*k* = −14→14
*T*_min_ = 0.755, *T*_max_ = 1.000	*l* = −9→8
8053 measured reflections	

### Refinement

**Table:**

Refinement on *F*^2^	Primary atom site location: structure-invariant direct methods
Least-squares matrix: full	Secondary atom site location: difference Fourier map
*R*[*F*^2^ > 2σ(*F*^2^)] = 0.014	Hydrogen site location: inferred from neighbouring sites
*wR*(*F*^2^) = 0.039	H-atom parameters constrained
*S* = 1.04	*w* = 1/[σ^2^(*F*_o_^2^) + (0.0188*P*)^2^ + 0.9351*P*] where *P* = (*F*_o_^2^ + 2*F*_c_^2^)/3
1841 reflections	(Δ/σ)_max_ = 0.002
110 parameters	Δρ_max_ = 0.42 e Å^−^^3^
0 restraints	Δρ_min_ = −0.25 e Å^−^^3^

### Special details

**Table:**

Geometry. All s.u.'s (except the s.u. in the dihedral angle between two l.s. planes) are estimated using the full covariance matrix. The cell s.u.'s are taken into account individually in the estimation of s.u.'s in distances, angles and torsion angles; correlations between s.u.'s in cell parameters are only used when they are defined by crystal symmetry. An approximate (isotropic) treatment of cell s.u.'s is used for estimating s.u.'s involving l.s. planes.
Refinement. Refinement of *F*^2^ against ALL reflections. The weighted *R*-factor *wR* and goodness of fit *S* are based on *F*^2^, conventional *R*-factors *R* are based on *F*, with *F* set to zero for negative *F*^2^. The threshold expression of *F*^2^ > 2σ(*F*^2^) is used only for calculating *R*-factors(gt) *etc*. and is not relevant to the choice of reflections for refinement. *R*-factors based on *F*^2^ are statistically about twice as large as those based on *F*, and *R*- factors based on ALL data will be even larger.

### Fractional atomic coordinates and isotropic or equivalent isotropic displacement parameters (Å^2^)

**Table:**

	*x*	*y*	*z*	*U*_iso_*/*U*_eq_	
I1	0.697282 (5)	0.925416 (9)	−0.003362 (13)	0.01546 (5)	
O1	0.70514 (6)	0.66598 (11)	−0.03589 (18)	0.0231 (3)	
O2	0.62939 (7)	0.52986 (10)	0.01236 (15)	0.0214 (3)	
O3	0.41118 (6)	0.68759 (8)	0.23820 (15)	0.0148 (2)	
N1	0.64607 (7)	0.63180 (12)	0.01188 (16)	0.0133 (3)	
C1	0.60441 (8)	0.83551 (12)	0.0734 (2)	0.0119 (3)	
C2	0.59202 (8)	0.71660 (12)	0.0719 (2)	0.0116 (3)	
C3	0.52698 (8)	0.67047 (12)	0.12652 (19)	0.0122 (3)	
H3	0.5199	0.5896	0.1236	0.015*	
C4	0.47214 (8)	0.74173 (12)	0.1854 (2)	0.0115 (3)	
C5	0.48273 (8)	0.86045 (12)	0.1870 (2)	0.0128 (3)	
H5	0.4455	0.9104	0.2254	0.015*	
C6	0.54812 (8)	0.90498 (12)	0.1319 (2)	0.0131 (3)	
H6	0.5548	0.9860	0.1342	0.016*	
C7	0.35196 (8)	0.75849 (13)	0.2943 (2)	0.0157 (3)	
H7A	0.3658	0.8025	0.4007	0.024*	
H7B	0.3107	0.7096	0.3228	0.024*	
H7C	0.3391	0.8116	0.1977	0.024*	

### Atomic displacement parameters (Å^2^)

**Table:**

	*U*^11^	*U*^22^	*U*^33^	*U*^12^	*U*^13^	*U*^23^
I1	0.01244 (8)	0.01476 (8)	0.01918 (8)	−0.00280 (4)	0.00198 (3)	0.00187 (4)
O1	0.0129 (6)	0.0209 (6)	0.0354 (7)	0.0015 (5)	0.0054 (5)	−0.0037 (5)
O2	0.0223 (7)	0.0104 (5)	0.0315 (7)	0.0033 (5)	0.0035 (5)	−0.0004 (4)
O3	0.0109 (5)	0.0121 (5)	0.0214 (6)	−0.0013 (4)	0.0029 (4)	−0.0001 (4)
N1	0.0128 (6)	0.0149 (6)	0.0124 (6)	0.0025 (5)	−0.0020 (4)	−0.0002 (5)
C1	0.0108 (7)	0.0137 (7)	0.0113 (7)	−0.0019 (6)	−0.0011 (5)	0.0018 (5)
C2	0.0122 (7)	0.0115 (6)	0.0111 (7)	0.0038 (6)	−0.0016 (5)	−0.0007 (5)
C3	0.0146 (7)	0.0091 (6)	0.0130 (7)	0.0003 (5)	−0.0024 (5)	0.0004 (5)
C4	0.0101 (6)	0.0135 (7)	0.0109 (7)	−0.0013 (5)	−0.0014 (5)	0.0007 (5)
C5	0.0116 (7)	0.0121 (6)	0.0148 (7)	0.0027 (6)	−0.0009 (5)	−0.0010 (5)
C6	0.0154 (7)	0.0097 (6)	0.0143 (7)	−0.0006 (6)	−0.0014 (6)	0.0003 (6)
C7	0.0110 (7)	0.0170 (7)	0.0191 (8)	0.0009 (6)	0.0019 (6)	0.0001 (6)

### Geometric parameters (Å, º)

**Table:**

I1—C1	2.1017 (14)	C3—C4	1.387 (2)
O1—N1	1.2238 (18)	C3—H3	0.9500
O2—N1	1.225 (2)	C4—C5	1.394 (2)
O3—C4	1.3574 (18)	C5—C6	1.386 (2)
O3—C7	1.4398 (18)	C5—H5	0.9500
N1—C2	1.4791 (19)	C6—H6	0.9500
C1—C6	1.395 (2)	C7—H7A	0.9800
C1—C2	1.4017 (19)	C7—H7B	0.9800
C2—C3	1.387 (2)	C7—H7C	0.9800
			
C4—O3—C7	117.44 (11)	O3—C4—C5	125.10 (13)
O1—N1—O2	122.90 (14)	C3—C4—C5	119.30 (13)
O1—N1—C2	119.00 (13)	C6—C5—C4	119.44 (13)
O2—N1—C2	118.10 (13)	C6—C5—H5	120.3
C6—C1—C2	116.72 (13)	C4—C5—H5	120.3
C6—C1—I1	114.66 (10)	C5—C6—C1	122.56 (13)
C2—C1—I1	128.62 (11)	C5—C6—H6	118.7
C3—C2—C1	121.54 (13)	C1—C6—H6	118.7
C3—C2—N1	115.27 (12)	O3—C7—H7A	109.5
C1—C2—N1	123.19 (13)	O3—C7—H7B	109.5
C2—C3—C4	120.42 (13)	H7A—C7—H7B	109.5
C2—C3—H3	119.8	O3—C7—H7C	109.5
C4—C3—H3	119.8	H7A—C7—H7C	109.5
O3—C4—C3	115.59 (12)	H7B—C7—H7C	109.5
			
C6—C1—C2—C3	−0.2 (2)	C7—O3—C4—C3	−177.84 (13)
I1—C1—C2—C3	−179.68 (11)	C7—O3—C4—C5	2.3 (2)
C6—C1—C2—N1	179.41 (13)	C2—C3—C4—O3	−179.07 (13)
I1—C1—C2—N1	−0.1 (2)	C2—C3—C4—C5	0.8 (2)
O1—N1—C2—C3	−178.97 (13)	O3—C4—C5—C6	179.05 (14)
O2—N1—C2—C3	1.04 (19)	C3—C4—C5—C6	−0.9 (2)
O1—N1—C2—C1	1.5 (2)	C4—C5—C6—C1	0.4 (2)
O2—N1—C2—C1	−178.54 (14)	C2—C1—C6—C5	0.1 (2)
C1—C2—C3—C4	−0.3 (2)	I1—C1—C6—C5	179.73 (12)
N1—C2—C3—C4	−179.93 (13)		

### Hydrogen-bond geometry (Å, º)

**Table:**

*D*—H···*A*	*D*—H	H···*A*	*D*···*A*	*D*—H···*A*
C7—H7*B*···O1^i^	0.98	2.58	3.4503 (19)	148

Symmetry code: (i) *x*−1/2, *y*, −*z*+1/2.
